# It’s Time to Shift the Paradigm: Translation and Clinical Application of Non-αvβ3 Integrin Targeting Radiopharmaceuticals

**DOI:** 10.3390/cancers13235958

**Published:** 2021-11-26

**Authors:** Susanne Kossatz, Ambros Johannes Beer, Johannes Notni

**Affiliations:** 1 Department of Nuclear Medicine, School of Medicine, Technical University of Munich, 81675 Munich, Germany; s.kossatz@tum.de; 2 Central Institute for Translational Cancer Research (TranslaTUM), School of Medicine, Technical University of Munich, 81675 Munich, Germany; 3 Klinik für Nuklearmedizin, Universitätsklinikum Ulm, 89081 Ulm, Germany; ambros.beer@uniklinik-ulm.de; 4 Department of Pathology, School of Medicine, Technical University of Munich, 81675 Munich, Germany; 5 TRIMT GmbH, 01454 Radeberg, Germany

**Keywords:** αvβ6-Integrin, radiopharmaceuticals, molecular imaging, translational medicine, pancreatic cancer, theranostics

## Abstract

**Simple Summary:**

Cancer cells often present a different set of proteins on their surface than normal cells. This also applies to integrins, a class of 24 cell surface receptors which mainly are responsible for physically anchoring cells in tissues, but also fulfil a plethora of other functions. If a certain integrin is found on tumor cells but not on normal ones, radioactive molecules (named tracers) that specifically bind to this integrin will accumulate in the cancer lesion if injected into the blood stream. The emitted radiation can be detected from outside the body and allows for localization and thus, diagnosis, of cancer. Only one of the 24 integrins, the subtype αvβ3, has hitherto been thoroughly investigated in this context. We herein summarize the most recent, pertinent research on other integrins, and argue that some of these approaches might ultimately improve the clinical management of the most lethal cancers, such as pancreatic carcinoma.

**Abstract:**

For almost the entire period of the last two decades, translational research in the area of integrin-targeting radiopharmaceuticals was strongly focused on the subtype αvβ3, owing to its expression on endothelial cells and its well-established role as a biomarker for, and promoter of, angiogenesis. Despite a large number of translated tracers and clinical studies, a clinical value of αvβ3-integrin imaging could not be defined yet. The focus of research has, thus, been moving slowly but steadily towards other integrin subtypes which are involved in a large variety of tumorigenic pathways. Peptidic and non-peptidic radioligands for the integrins α5β1, αvβ6, αvβ8, α6β1, α6β4, α3β1, α4β1, and αMβ2 were first synthesized and characterized preclinically. Some of these compounds, targeting the subtypes αvβ6, αvβ8, and α6β1/β4, were subsequently translated into humans during the last few years. αvβ6-Integrin has arguably attracted most attention because it is expressed by some of the cancers with the worst prognosis (above all, pancreatic ductal adenocarcinoma), which substantiates a clinical need for the respective theranostic agents. The receptor furthermore represents a biomarker for malignancy and invasiveness of carcinomas, as well as for fibrotic diseases, such as idiopathic pulmonary fibrosis (IPF), and probably even for Sars-CoV-2 (COVID-19) related syndromes. Accordingly, the largest number of recent first-in-human applications has been reported for radiolabeled compounds targeting αvβ6-integrin. The results indicate a substantial clinical value, which might lead to a paradigm change and trigger the replacement of αvβ3 by αvβ6 as the most popular integrin in theranostics.

## 1. Introduction

**Figure 1:**
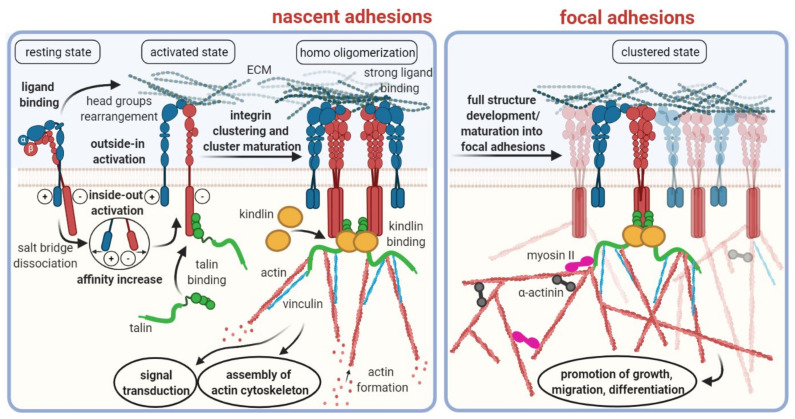
Schematic overview of integrin structures and functions. Note that this scheme illustrates only the processes involved into integrin binding to the extracellular matrix. Other important functionalities of certain integrins, such as TGF-β activation (see below), are not represented herein. Copyright notice: Figure reprinted from Cancers 2021, 13, 1711. Ludwig et al., RGD-Binding Integrins Revisited: How Recently Discovered Functions and Novel Synthetic Ligands (Re-). Shape an Ever-Evolving Field [5]; under Creative Commons CC BY 4.0.

Integrins are a family of cell surface receptors which primarily mediate the binding and physical attachment of cells to various insoluble strand proteins, such as collagen, laminin, fibronectin, vitronectin, and others, that constitute the extracellular matrix (ECM) [1]. As such, they fulfil a pivotal role for tissue connectivity and stiffness [2]. Integrins are transmembrane proteins, i.e., they possess an extracellular domain for binding to their ligands, and an intracellular domain which is mostly connected to the actin filaments forming the cytoskeleton (Figure 1). The intracellular domain is furthermore involved in signaling cascades [3]. On the one hand, factors in the cytosol can induce activation and clustering of integrins (inside–out signaling); on the other hand, ligand binding to integrins can initiate intracellular processes (outside–in signaling), thus underscoring the multitude of biological functions attributed to integrins in general [4].

**Figure 2:**
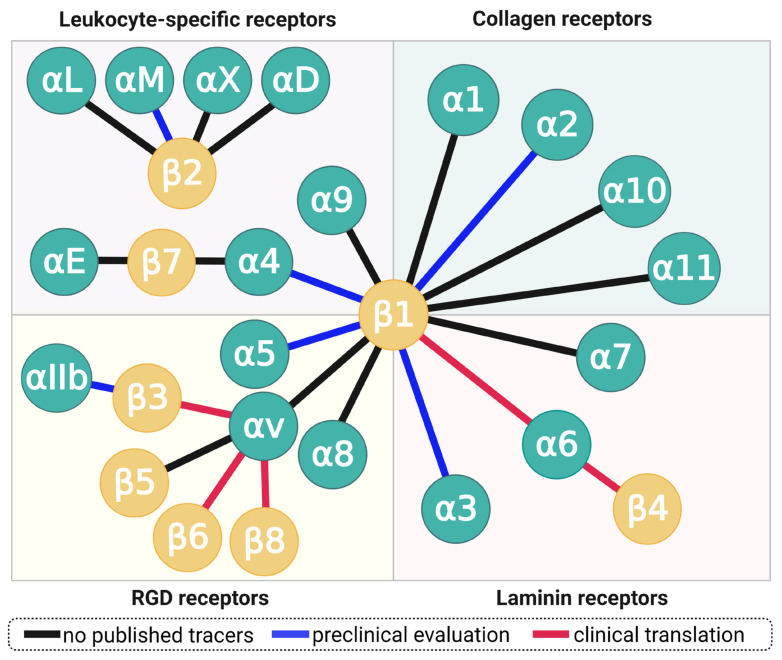
The family integrin receptors. Each connecting line represents an integrin, formed by dimerization of one α- and one β-subunit. Blue lines indicate integrins for which radiolabeled small-molecule ligands, peptides, or peptidomimetics have been characterized in small animals; red lines denote clinical translation, regardless of cohort size (i.e., including single-case applications). Copyright notice: Figure reprinted from EJNMMI Res. 2021, 11, doi:10.1186/s13550-021-00842-2. Steiger et al., There is a world beyond αvβ3-integrin: Multimeric ligands for imaging of the integrin subtypes αvβ6, αvβ8, αvβ3, and α5β1 by positron emission tomography [6]; under Creative Commons CC BY 4.0.

Each integrin is formed by dimerization of two distinct subunits, one α- and one β-chain. 18 different α- and 8 β-proteins have been discovered so far, but not all combinations are capable of pairing—a total of 24 different combinations is known today (Figure 2). Ligand binding occurs on the most distant extracellular contact site of both subunits, where a protein-bound zinc(II) ion and a more or less hydrophobic pocket form a distinct, unique recognition motif stretching across both subunits. Each of the 24 dimers therefore has its own, unique affinity profile and can, for example, specifically recognize a single ECM protein, or bind to a broader range of substrates. It is worth emphasizing that each of the 26 integrin subunits is genetically encoded, transcribed, and translated independently. Studies addressing the expression and functions of a particular integrin therefore require, for example, the determination of mRNA and/or cytosolic concentration of both comprising subunits, or the direct detection of the actual dimer. This implicates that the abundance of a particular subunit, e.g., β1, does not determine any functionality or property of one of its respective dimers, e.g., αvβ1, α5β1, et cetera. On the other hand, the lack of a particular subunit may have specific and well-defined consequences due to a loss of all respective dimers, which is why many researchers actually investigated the impact of knockout of a certain ITG gene to conclude on the respective dimers’ functions from the resulting malfunctions observed in mice.

Certain integrin subunits are capable of forming a multitude of dimers, most notably, β1, which dimerizes with 12 different α-chains, and αv which pairs with β1, β3, β5, β6, and β8 (Figure 2). Although all αv integrins (plus α5β1, α8β1, and αIIbβ3) recognize the short tripeptide sequence arginine–glycine–aspartate (RGD), they nevertheless are expressed by different cell types and, in part, bind to proteins with different structures and completely different biological functions. For example, α5β1-integrin is expressed by activated endothelial cells, binds to the ECM protein fibronectin, and regulates angiogenesis [7]—whereas the dimer αvβ6 is exclusively expressed by epithelial cells and releases transforming growth factor beta (TGF-β) [8] by snatching and physically deforming latency associated peptide (LAP), the “protective envelope” that wraps up TGF-β while lounging in the interstitium (vide infra) [9]. Both integrins exert their functions by binding to an RGD motif in their respective substrates, which clearly demonstrates that RGD-recognizing integrins neither necessarily share the same class of molecular targets or biochemical purpose, nor does presence of the RGD motif in recognized proteins implicate a similar biological function or mechanism [5].

That being said, it appears worthwhile to have a closer look at the common perception of integrins and their ligands in the context of molecular imaging and radiopharmacy [6]. An in-depth look at the literature of the last 1–2 decades reveals that the overwhelming majority of studies has been conducted using (radio)labeled ‘RGD peptides’ [10,11,12]. This term mostly refers to cyclic pentapeptides of the cyclo(RDGxX) type [13] which are often abbreviated as ‘cRGD’ and possess a high affinity and selectivity for αvβ3-integrin [14] (x = d-Phe, d-Tyr; X = Val, Lys, Glu; conjugation and/or labeling is usually done on the Lys or Glu side chains). Since αvβ3 was the first integrin shown to be closely associated with angiogenesis [15,16], most of these investigations aimed at targeting angiogensis in various settings and diseases—above all, in cancer [17,18,19] but also in arthritis, wound healing, and other instances of medical interest.

The narrative of targeting αvβ3-integrin for imaging of angiogenesis using (radio) labeled ‘RGD peptides’ became so popular that the terms ‘αvβ3’, ‘RGD’, and ‘angiogenesis’ were (and still are) frequently used in an almost synonymous manner, such as in the phrase “angiogenesis imaging using Ga-68-RGD PET/CT” [20]. A strict causal relationship, however, does not exist for any combination of these terms. For example, neither the αv- [21] nor the β3-subunit [22] (and, therefore, αvβ3) is strictly required for angiogenesis. A lack of β3 can, for example, be compensated by up-regulation of integrin-independent angiogenic pathways, such as vascular endothelial growth factor receptor 2 (VEGFR2) signaling [23]. αvβ3 is not only expressed on activated endothelial cells but also on many tumor cells and on macrophages [24]. It was shown recently that homing of cRGD-decorated nanocarriers to tumor vasculature may also occur by action of phagocytes, substantiating that targeting angiogenic sites with cRGD-containing constructs may involve other mechanisms than straightforward binding to endothelial αvβ3-integrin [25]. Moreover, there are selective ligands for αvβ3-integrin which do not rely on the RGD motif [26,27]. Vice versa, many (cyclic) peptides have been described which contain the RGD sequence but do not preferably bind to αvβ3 but another integrin, such as αvβ5, αvβ6, αvβ8, or α5β1 [5]. The “classical” cRGD pentapeptides [13], i.e., c(RGDxK) and its congeners (see above), are nonetheless selective for αvβ3 [28]; a fact which is sometimes disregarded and leads to confusion about the integrins and biological structures targeted by the respective conjugates [29].

During the last two decades, translational and clinical studies involving integrin-targeted radiopharmaceuticals were nonetheless focused almost exclusively on αvβ3-integrin ligands based on cyclic RGD peptides [12,30]. Their overwhelming popularity may have shaped and defined the general opinion about integrin tracers and their clinical potential. In spite of a large number of clinical studies and an even larger number of synthesized tracers, a well-defined clinical application of αvβ3-integrin targeted compounds could not be established throughout this time [31]. A broad clinical application or even a breakthrough similar to prostate-specific membrane antigen (PSMA) targeted agents [32] never occurred, and this is also quite improbable to happen in the future, in view of the substantial efforts already made [30]. A certain degree of resignation and a comparably low level of interest in integrin-targeted radiopharmaceuticals within the radiopharmaceutical and nuclear medicine communities thus comes as no surprise. We nonetheless believe that integrins could turn out to be valuable theranostic targets after all—on the condition that a broader view is established and pertinent research will continue off the beaten tracks. This review will thus summarize the latest reports on clinical translation of radiolabeled ligands for integrins other than αvβ3, in order to shed light on the untapped potential and the hidden treasures offered by this multifaceted class of receptors. A brief glance on αvβ3-integrin driven theranostics and the associated challenges is nonetheless included as well.

## 2. αvβ3-Integrin—A Cul-De-Sac?

The above-mentioned availability of cRGD peptides as versatile ligands for αvβ3-integrin [33] and the knowledge about the importance of this receptor for a highly relevant biological mechanism—angiogenesis—triggered the development of the respective radiolabeled derivatives [34,35] and the first clinical application of an αvβ3-directed PET tracer, [^18^F]Galacto-RGD [36,37,38]. Other, similar tracers followed shortly thereafter, and clinical studies were conducted to establish the novel class of radiopharmaceuticals for cancer imaging [39,40,41], more or less explicitly seeking to augment or even replace [^18^F]2-fluoro-2-deoxyglucose (FDG). Many of these investigations also aimed to exploit the fact that αvβ3-integrin is frequently expressed by tumor cells, not only endothelial cells involved in angiogenesis.

**Figure 3:**
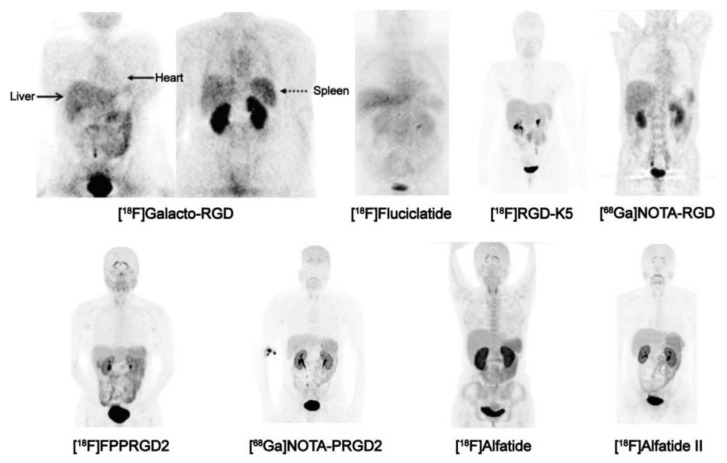
Biodistribution of PET imaging agents based on RGD peptides, 1 h p.i., in healthy human subjects (except [^18^F]Galacto-RGD, which shows a patient with osteomyelitis). Images in the upper row were obtained with monomeric agents (i.e., comprising only one RGD per molecule), the bottom row with dimers (two RGD per molecule). Note the typical pattern of physiological uptake in liver, spleen, intestines, thyroid, and plexus choroideus, arising from low-level αvβ3-integrin expression in these organs. The pattern is more clearly discernible for the dimers (bottom row), because their generally higher affinity [45] causes a higher sensitivity and thus, a higher uptake in tissues with low αvβ3 expression density. Strong signals in kidneys and urinary bladder are caused by renal excretion of all tracers. Copyright notice: Figure reprinted from Theranostics 2016;6:78–92. Chen et al., Clinical Application of Radiolabeled RGD Peptides for PET Imaging of Integrin αvβ3 [30]; under Creative Commons CC BY 4.0.

Triggered by the early success of ‘theran(g)ostics’, above all, the application of imaging agents in tandem with particle-emitting nuclides for diagnosis and endoradiotherapy of somatostatin-receptor 2 (SSTR2) positive neuroendocrine tumors (NET) [42], it appeared logical and obvious to adapt the same scheme to radiolabeled RGD peptides. In practice, this turned out to be more complicated than expected, because compared to SSTR2-expression in NET, αvβ3-integrin expression showed a higher degree of interindividual variation for most tumor entities, and αvβ3-integrin dependent uptake in target-positive lesions is usually substantially lower as compared to SSTR2-mediated accumulation of radiopharmaceuticals [43,44]. Target-to-organ ratios are furthermore lower because αvβ3-integrin is physiologically expressed to a certain extent in various organs and tissues (Figure 3) [30,36].

Targeted radionuclide therapy using popular radiometals like ^177^Lu, ^90^Y, or ^225^Ac [46] therefore bears a considerable risk of critical off-target doses and, therefore, radiotoxicity. These circumstances might eventually have impeded a notable clinical impact; at least, we are not aware of a broad clinical application of αvβ3-integrin targeted radionuclide therapeutics, regardless of compound structure or radionuclide. The same applies for tumor imaging with RGD-peptide based PET or SPECT imaging agents. In view of the fact that many attempts have been made but apparently none resulted in a broad clinical success or the approval of a pertinent radiopharmaceutical by the FDA or the EMA, we conclude that αvβ3-integrin is almost certainly not an optimal target for radionuclide theranostics.

However, we believe that there is still a considerable potential of αvβ3-integrin imaging, provided the study design of future clinical trials goes beyond the popular “if you can see it, you can treat it” theranostic approach. αvβ3-Integrin has not only been suggested as a predictive marker for many cancers [5,19,47], but also for non-oncological conditions, such as evaluation of cardiac remodeling [48,49,50] and plaque vulnerability [51,52], or rheumatoid arthritis [53,54]. Future studies seeking to establish clinical applications for αvβ3-integrin PET might therefore be successful if they are based on a robust biochemical rationale that truly reflects the complexity of αvβ3-dependent processes, and address a real clinical need, i.e., enable an improved, image-based clinical decision making for a relevant medical question [47].

## 3. αvβ6-Integrin—A Rising Star?

### 3.1. Relevance of αvβ6-Integrin for TGF-β Activation, Fibrosis, and Carcinoma Invasiveness

Other than αvβ3, αvβ6-integrin is exclusively expressed by epithelial cells [55]. The search for ligands targeting this integrin has been pursued already for a long time because of its key function as an activator of transforming growth factor β (TGF-β) [56]. TGF-β, in turn, is a pleiotropic cytokine which is highly conserved across species and produced by virtually all mammalian cells. TGF-β is involved in a large number of human pathologies [57], rendering it a highly relevant pharmacological target and, as such, a subject of intense research [58]. Most importantly, TGF-β is a universal growth suppressor which regulates gene transcription, DNA duplication and, thus, cell proliferation (mitosis) via smad-dependent signaling—not only for resident cells but also immune cells, such as leukocytes. As such, it is essential for tissue homeostasis, suggesting that pathologically low systemic TGF-β levels may be associated with autoimmune diseases and a higher probability of developing cancer. TGF-β overabundance or a loss of essential components in the respective signaling cascade may therefore trigger a broad variety of disease morphologies, such as fibrosis, atherosclerosis, and tumorigenesis, as well as tumor invasion and metastasis [57].

**Figure 4:**
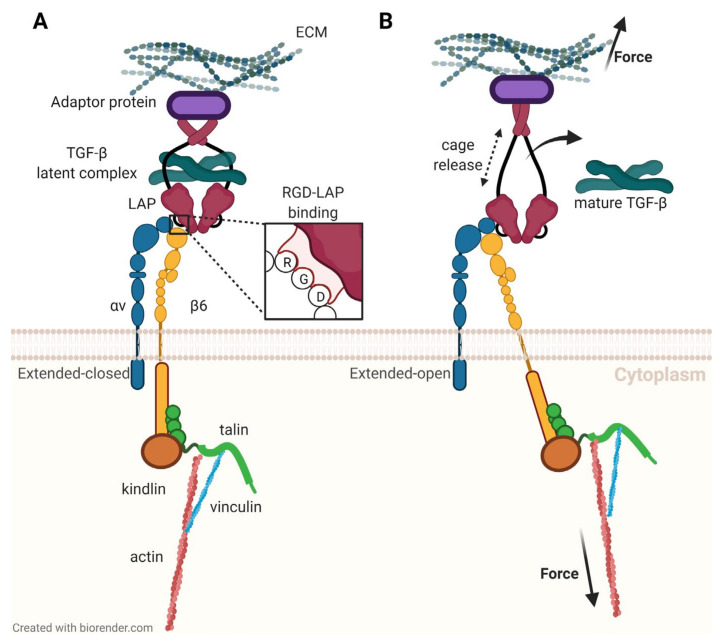
Activation of latent transforming growth factor β (TGF-β) by interaction of αvβ6-integrin with latency-associated peptide (LAP), according to a study by Dong et al. [61]. (**A**): αvβ6-integrin binds to an RGD motif in LAP. (**B**): A pulling force transmitted by the β6-subunit deforms LAP and releases TGF-β.

However, TGF-β is not secreted by cells into the interstitium in its active form (i.e., freely diffusible and able to bind to its receptors). Cells rather produce and sequester a complex with another protein called latency-associated peptide (LAP) [59]. Briefly, this aggregate named small latent complex (SLC) is linked to the ECM by another protein referred to as latent TGF-β binding protein (LTBP), and TGF-β must be released from the entire prodomain, called large latent complex (LLC), to take action. This activation is a key functionality of αvβ6-integrin [60]. Figure 4 illustrates that on a molecular level, this process occurs by binding of the integrin to an RGD motif in LAP; then, a pulling force is exerted via the β6-subunit which is intracellularly bound to the actin filaments forming the cytoskeleton [61]. The force changes the structure of the ‘envelope’ LAP, the inter-protein binding forces are weakened, and TGF-β is released [9].

Overexpression of αvβ6-integrin therefore endows cells with the capability to enhance TGF-β levels in their vicinity, which is why its expression is tightly linked to diseases associated with TGF-β dysregulation. Assuming that, e.g., tumor cells have become insensitive to the antiproliferative signaling of TGF-β due to loss of smad4 [62] or p53 [63], a high TGF-β concentration actually promotes their proliferation and invasion of the surrounding tissue [64], because it suppresses the growth of normal cells, suppresses the immune system’s antitumor activity [65], triggers epithelial–mesenchymal transition (EMT) [66], and promotes angiogenesis. In accordance with this notion, several immunohistochemical studies have confirmed a high level of αvβ6-integrin at the infiltrative margins of epithelial cancers, particularly on carcinoma cells invading the stroma [6,67,68].

Since a therapeutic intervention by systemic inhibition of TGF-β may cause the aforementioned complications associated with low systemic TGF-β [69,70], the targeting of its activators, e.g., αvβ6-integrin [71,72], has been suggested as a more site-specific approach [47,67,73]. This strategy is currently pursued for treatment of fibrosis, a class of diseases associated with elevated TGF-β signaling [58] and αvβ6-integrin expression [74]. Rather than a systemic TGF-β blockade with, e.g., antibodies, an anti-fibrotic therapy using a small-molecule αvβ6-integrin inhibitor has been adapted successfully for control of pulmonary fibrosis [75]. αvβ6-Inhibitors have furthermore been suggested as cancer therapeutics [71,76], above all, for pancreatic cancer [72,77], which is characterized by a particularly high αvβ6-integrin expression rate. An investigation of nearly 400 patient specimens of pancreatic ductal adenocarcinoma (PDAC) revealed β6 positivity in 88% of primaries, in virtually lymph node and distant metastases—and also in many of its immediate precursor lesions, grade 3 pancreatic intraepithelial neoplasia (PanIN3) [78]. The integrin has furthermore been found in high density in many other carcinomas [55], like head-and-neck squamous cell (HNSCC), lung adeno (NSCLC), ovarian, and others, and furthermore has a prognostic value for some heterogeneous entities, for example, colorectal carcinoma (CRC) [79]. Finally, αvβ6-integrin might be a target of interest in the context of COVID-19 [80].

### 3.2. Towards Clinical Application of αvβ6-Integrin Imaging

The described biochemical and clinical background points at an urgent need for αvβ6-integrin imaging agents, specifically PET tracers, because the high intrinsic sensitivity and resolution of PET could potentially deliver highly valuable information to advance the clinical management of the aforementioned diseases. This encompasses not only initial diagnostics of cancers and fibrosis based on PET imaging. A personalized management of αvβ6-integrin targeted therapies, e.g., the image-based patient stratification, the early assessment of the efficacy of anti-fibrotic or anti-cancer drugs in order to adapt and improve dosage schemes, or the early identification of non-responders to save patients from a therapy’s side effects by discontinuation, might eventually generate an even higher clinical impact [47]. The search for αvβ6-integrin targeted tracers has therefore been initiated quite some time ago [19].

Hausner and colleagues from UC Davis pioneered this field with a report on an ^18^F labeled αvβ6-integrin binding peptide named A20FMDV (sequence: NAVPNLRGDLQVLAQKVART) and its use for PET imaging of tumor xenografts in mice [81]. The same group continued with preclinical optimization of the radiolabeled A20FMDV derivatives, e.g., by examining the effect of PEG28-linkers on both termini of the peptide [82,83], and investigated other labeling approaches, e.g., fluorination by means of strain-promoted click chemistry (SPAAC) [84] or AlF chemistry [85]. They ultimately selected the best-performing candidate for translation, which was [^18^F]fluorobenzoyl-PEG28-A20FMDV-PEG28, renamed it to [^18^F]αvβ6-BP (an abbreviation of [^18^F]αvβ6 binding peptide), and tested it for PET imaging of lung adenocarcinoma and breast carcinoma in humans [86]. The same compound was used for imaging of lung fibrosis in humans [87] and subsequently for monitoring the therapeutic effect of an inhaled small-molecule αvβ6-integrin inhibitor named GSK3008348 [88], and recently tested for PET/CT of SARS-CoV-2 infection related lesions in the lung [89].

Another approach was pursued by Kimura and colleagues from Stanford, who developed a series of selective αvβ6-integrin ligands based on an engineered peptide knot scaffold with three cysteine bridges, referred to as cysteine knot peptides or ‘knottins’ [90,91,92]. Eventually, ^68^Ga and ^18^F-labeled PET tracers based on one of the peptides, referred to as R_0_1-MG, were characterized in tumor-xenografted mice, and utilized for imaging of pancreatic, cervical and lung cancer, as well as of idiopathic pulmonary fibrosis (IPF) [93].

Researchers from Heidelberg University (Germany) exploited a phage-display engineering approach on an established cysteine-bridged scaffold, the sunflower trypsin inhibitor. They developed two αvβ6-integrin targeted peptides named SFITGv6 (sequence: GRCRFRGDLMQLCYPD) [94] and SFLAP3 (sequence: GRCTGRGDLGRLCYPD) [95]. Both peptides were conjugated to DOTA and labeled with ^68^Ga, and used for PET-imaging of patients with HNSCC [95], hypopharynx tumor, [94], NSCLC [94,96], and pancreatic cancer [97].

Researchers from Beijing recently introduced ^68^Ga-cycratide, a ^68^Ga-labeled DOTA conjugate of a cyclic peptide with the sequence c(RGDLATLK). They reported in-vitro as well as preclinical data in tumor-bearing mice, and, furthermore, performed PET imaging in five healthy volunteers and two pancreatic cancer patients [98].

At TU Munich (Germany), Kessler and coworkers developed a class of cyclic nonapeptides with a high αvβ6-integrin selectivity and metabolic stability [99]. The most selective structure with the sequence c(FRGDLAFp(*N*Me)K) was conjugated to the ^68^Ga chelator TRAP [100] by means of Cu^II^-catalyzed azide–alkyne cycloaddition (CuAAC) [101,102], giving rise to monomeric, dimeric, and trimeric conjugates [103]. A preclinical characterization in tumor-xenografted mice revealed that the multimers exhibited extraordinarily high affinities (IC_50_ of 260 and 23 pM for the monomer and the trimer, respectively). The trimer unfortunately showed a high degree of nonspecific uptake in the bowel organs (intestines and liver), whereas various monomeric conjugates showed insufficient tumor accumulation [104]. A revision of the peptide sequence finally led to the development of Trivehexin, a TRAP-based trimer of c(YRGDLAYp(*N*Me)K) [105]. ^68^Ga-Trivehexin was subsequently tested for imaging of patients with PDAC [106] as well as HNSCC and adenocarcinoma of the parotid duct [105].

### 3.3. Clinical αvβ6-Integrin PET for Cancer Imaging

To the best of our knowledge, no more than the above-mentioned, radiolabeled αvβ6-integrin specific ligands have been investigated in humans so far, although other highly promising radiotracers have been synthesized and characterized preclinically [107,108,109,110]. The already published clinical data nonetheless allow for a glance into the future and illustrate the potential of this class of PET imaging agents.

**Figure 5:**
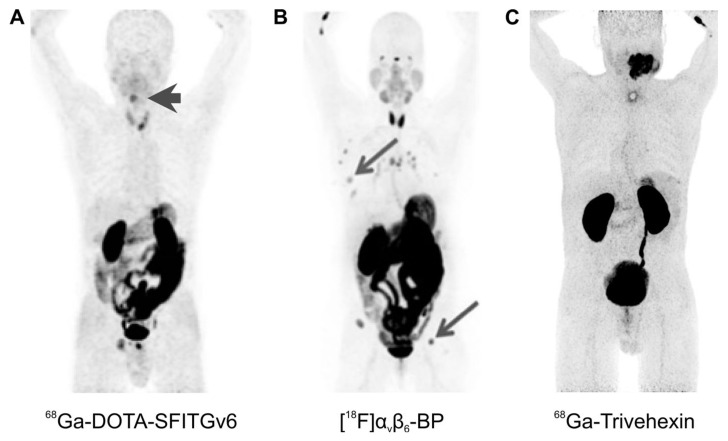
αvβ6-integrin PET imaging in cancer patients (maximum intensity projections, 1 h p.i.) with different radiopharmaceuticals; arrows (wherever shown) indicate tumor lesions. (**A**): ^68^Ga-DOTA-SFITGv6 PET of a recurrent hypopharynx tumor. (**B**): A stage IV invasive mammary carcinoma, imaged using [^18^F]α_v_β_6_-BP. (**C**): ^68^Ga-Trivehexin PET of a large oral squamous cell carcinoma. Copyright notice: Images were adapted and reprinted from: (**A**) Clin. Cancer Res. 2017, 23, 4170–4180. Altmann et al., Identification of a Novel ITGαvβ6-Binding Peptide Using Protein Separation and Phage Display [94]; and (**B**) Clin. Cancer Res. 2019, 25, 1206–1215; Hausner et al., Preclinical Development and First-in-Human Imaging of the Integrin αvβ6 with [^18^F]αvβ6-Binding Peptide in Metastatic Carcinoma [86]; with permission from AACR and not included in CC-BY 4.0 of this article. (**C**) Eur. J. Nucl. Med. Mol. Imaging 2021, doi:10.1007/s00259-021-05559-x. Quigley et al., PET/CT imaging of head-and-neck and pancreatic cancer in humans by targeting the “Cancer Integrin” αvβ6 with Ga-68-Trivehexin [105]; under Creative Commons CC BY 4.0.

**Figure 6:**
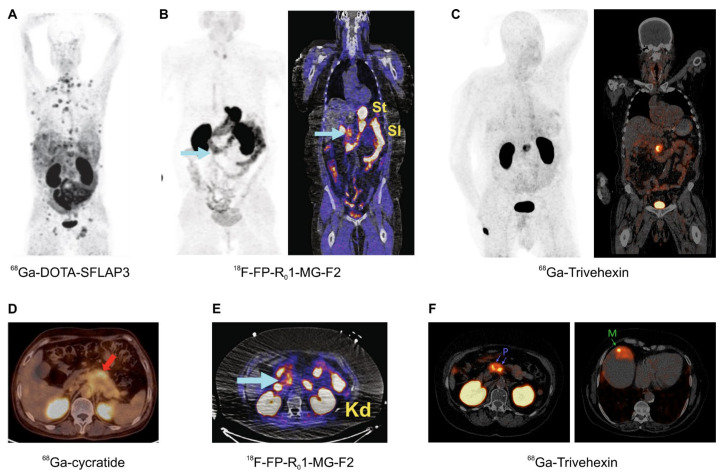
αvβ6-integrin PET/CT imaging in pancreatic cancer (MIPs (**A**–**C**), coronal slices (**B**,**C**), and axial slices (**D**–**F**); (**A**–**E**), 1 h p.i.; F, 2 h p.i.); arrows (wherever shown) indicate tumor lesions. (**A**): Highly metastatic pancreatic carcinoma, imaged using ^68^Ga-DOTA-SFLAP3. (**B**,**E**): MIP and fusion images of a non-metastatic pancreatic carcinoma, obtained using the knottin peptide based tracer ^18^F-FP-R_0_1-MG-F2. (**C**,**F**): ^68^Ga-Trivehexin PET/CT of a non-metastatic (**C**) and metastatic (**F**) PDAC (“P” indicates the primaries, “M” a liver metastasis). (**D**): Axial fusion PET/CT of a pancreatic cancer obtained with ^68^Ga-cycratide. Copyright notice: Images were adapted and reprinted from (**A**) Nuklearmedizin 2019, 58, 309–18. Müller et al., Preclinical evaluation of peptide-based radiotracers for integrin αvβ6-positive pancreatic carcinoma. [97] © Georg Thieme Verlag KG. (**B**,**E**) Nat. Commun. 2019, 10, 4673. Kimura et al., Evaluation of integrin αvβ6 cystine knot PET tracers to detect cancer and idiopathic pulmonary fibrosis [93]; (**C**) Eur. J. Nucl. Med. Mol. Imaging 2021, 48, 4107–4108. Quigley et al., PET/CT imaging of pancreatic carcinoma targeting the “cancer integrin” αvβ6 [106]; and (**F**) Eur. J. Nucl. Med. Mol. Imaging 2021, doi:10.1007/s00259-021-05559-x. Quigley et al., PET/CT imaging of head-and-neck and pancreatic cancer in humans by targeting the “Cancer Integrin” αvβ6 with Ga-68-Trivehexin [105]; under Creative Commons CC BY 4.0. (**D**) This research was originally published in JNM. Feng et al., Clinical Translation of a ^68^Ga-Labeled Integrin α_v_β_6_–Targeting Cyclic Radiotracer for PET Imaging of Pancreatic Cancer. J. Nucl. Med. 2020;61:1461–1467. [98] © SNMMI.

Overall, the hitherto translated PET tracers show predominantly renal clearance; hence, a strong signal is invariantly observed in the kidneys and the urinary bladder (Figure 5 and Figure 6). In addition, some of the agents show more or less prominent non-specific uptake in several organs, most notably, in stomach and intestines. Figure 5 demonstrates that αvβ6-integrin imaging might nevertheless be suitable for visualization of a variety of cancers. This is in accordance with a solid body of evidence that this integrin is expressed in many carcinomas [55]. A particularly high-expression density and, therefore, a high diagnostic relevance of pertinent imaging agents, has been suggested for HNSCC [55]. In line with this notion, a good tumor delineation could be observed for a large sublingual carcinoma using ^68^Ga-Trivehexin (Figure 5C), and for a hypopharynx tumor using ^68^Ga-DOTA-SFITGv6 (Figure 5A).

The most promising field of application arguably is the imaging of pancreatic cancer, because PET imaging of this entity is not reliably possible with the standard tracer [^18^F]FDG [111]. This applies all the more because PDAC is one of the carcinomas with the worst prognosis, and treatment options are still limited as compared to other malignant cancers, such as prostate carcinoma. A powerful agent for imaging of PDAC might therefore not only be important for diagnostics, e.g., to improve planning of surgery, but also implicates a perspective for future development of targeted radiotherapeutics. An overview of αvβ6-integrin targeted PET/CT with different tracers (Figure 6) corroborates the feasibility of this approach, not only for imaging of PDAC primaries but also of small liver metastases thereof (Figure 6F).

The hitherto reported clinical αvβ6-integrin PET data comprise only a few cases and thus, limited conclusions can be drawn at this stage. The available images of cancers nonetheless clearly indicate a high potential and might, therefore, boost the research in this field, and, above all, trigger the development of the respective radiotherapeutics labeled with the commercially available beta emitter ^177^Lu, or emerging alpha emitters like ^225^Ac [46]. This, in turn, is a hopeful perspective for many of pancreatic cancer patients who, in view of a very short overall survival after diagnosis, are in desperate need for novel, improved therapies.

## 4. αvβ8-Integrin PET—A Solution without a Problem?

The integrin subunit β8 was discovered as the last among the five β subunits which exclusively dimerize with αv [112]. αvβ8-Integrin appears to be the odd one out, as considerably less is known about this dimer than about the other αv integrins [5,19]. It is predominantly expressed on astrocytes and, just like αvβ6, is an activator of TGF-β, although by a different mechanism. In contrast to αvβ6-integrin which releases TGF-β by exerting a pulling force on LAP (vide supra), αvβ8 also recognizes and deforms LAP, but without cleaving the SLC. Instead, the binding of αvβ8 leads to activation of TGF-β without releasing it from the LAP, by exposing the receptor-binding sites of TGF-β [113]. Irrespective of the activation mode, one would thus expect comparable diagnostic and therapeutic implications for αvβ6 and αvβ8, i.e., a strong connection to TGF-β-driven disease patterns. Expression and functions of αvβ8-integrin in cancer and fibrosis has been discussed as well, but there is less evidence for a clinical relevance than in the case of αvβ6-integrin [19]. A high proportion of β8 positive tumor cells was recently detected in various carcinomas (ovarian, uterine endometrioid, skin, in situ breast ductal, gastric adenocarcinoma, and, particularly, oral squamous cell carcinoma) by histological methods, but the small numbers of patient specimens (3–22 per entity) still leave some doubt whether αvβ8-integrin could actually be useful for cancer imaging or theranostics [114]. A very intriguing discovery was recently made by Takasaka and colleagues, who found that up-regulation of αvβ8-integrin is apparently another mechanism for immune evasion of tumor cells. Blockade of this integrin has been shown to potentiate a cytotoxic T cell response in tumors, independently of the PD1–PD-L1 axis, thus suggesting αvβ8-integrin as a target for immune checkpoint therapy [114].

Since the wealth of information about αvβ3-integrin is most likely a result of the long-term availability of small-molecule inhibitors, i.e., cRGD peptides [10], the comparably low amount of knowledge about αvβ8-integrin might, likewise, mainly be a result of a lack of respective ligands. This situation has changed recently with the discovery of a small, selective, and stable ligand for αvβ8 [115]. The cyclic octapeptide with the sequence c(GLRGDLp(*N*Me)K) was subsequently—in analogy to the above-mentioned ^68^Ga-Trivehexin—trimerized on the TRAP chelator scaffold, in order to generate a practicable PET radiopharmaceutical named ^68^Ga-Triveoctin [116]. Trimerization [6] again greatly increased the affinity, and the encouraging preclinical results prompted a clinical translation.

**Figure 7:**
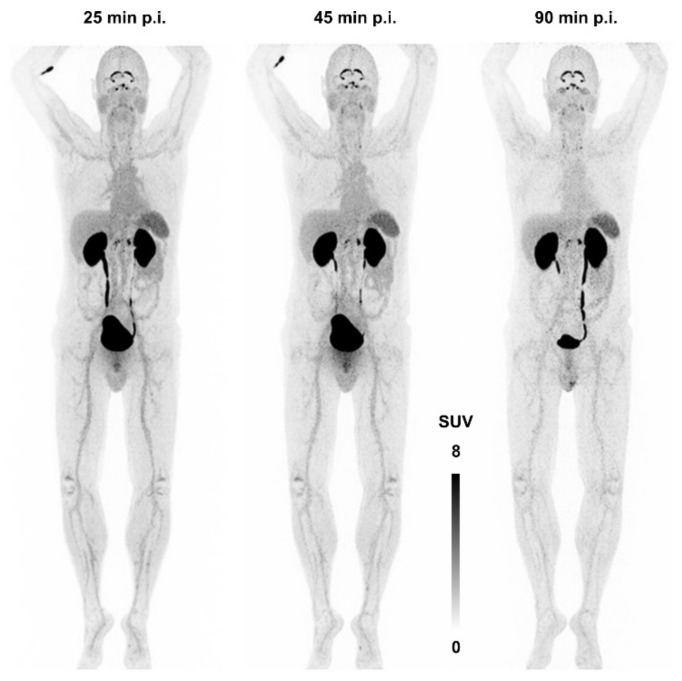
αvβ8-integrin PET imaging (maximum intensity projection) in human using ^68^Ga-Triveoctin. Copyright notice: Figure reprinted from EJNMMI Res. 2020, 10, 133. Quigley et al., Tracking a TGF-β activator in vivo: sensitive PET imaging of αvβ8-integrin with the Ga-68-labeled cyclic RGD octapeptide trimer Ga-68-Triveoctin [116]; under Creative Commons CC BY 4.0.

Figure 7 shows the first—and, currently, the only—αvβ8-integrin PET image acquired in human. There is apparently neither a strong specific nor a non-specific uptake in any major organ, apart from a strong signal in kidneys and bladder due to renal excretion. The notion of β8-integrin expression on neural cells (astrocytes) might nonetheless be connected to a substantial uptake in the plexus choroideus and -coeliacus [115]. However, due to a lack of pertinent immunohistochemistry data, no definitive statement can currently be made concerning this observation, and any conclusion would be premature. This situation is quite typical for the entire field of αvβ8-integrin imaging—many hypotheses are not yet supported with enough data and experience. It is nevertheless expected that the knowledge about the in vivo expression patterns of this integrin will substantially grow in the near future, because an imaging tool is now available.

## 5. Translation of Radiopharmaceuticals Targeting Other Integrins

The largest part of radioligands was reported for the integrins discussed above, and comparably few attempts have been made to establish other integrins as targets for radio-theranostics. These include the laminin receptors α6β1, α6β4, and α3β1 [117], the collagen receptor α2β1 [118], as well as the leucocyte-specific receptors α4β1 [119,120,121] and αMβ2 [122,123]. The laminin receptors α6β4 and α6β1 arguably have the largest potential next to RGD receptors, because they are expressed by several cancers such as colorectal, squamous cell, small cell lung, breast, pancreatic, and prostate carcinoma, while their expression has been linked to poor survival [124]. α6β4 signaling in malignant cells cooperates with growth factor receptors and amplification of well-known tumor-promoting pathways, such as PI3K, AKT, and MAPK, thus resulting in, for example, proliferative signaling, tumor invasion, metastasis, evasion of apoptosis, and stimulation of angiogenesis. Expression of α6-integrin in general, and the dimer α6β1 in particular, has been linked to poor prognosis of breast cancer and hepatocellular carcinoma [125].

An ^18^F-labeled derivative of the peptide sequence CRWYDENAC (referred to as RWY) was reported as the first α6-integrin PET tracer and preclinically characterized in mouse models of hepatocellular cancer [126]. Of note, this peptide is apparently not selective for a single integrin but binds to both α6β4 and α6β1, which somewhat limits the strength of the rationale required for a meaningful interpretation of in vivo signals. It can be assumed that selective targeting has not yet been achieved due to structural similarity of both integrins’ binding sites and/or the conformational flexibility of the linear amino acid sequence. This peptide was nonetheless applied for first-in-human imaging in form of the monomeric SPECT tracer ^99m^Tc-RWY, which was able to clearly delineate tumor lesions in two breast cancer patients [127]. Compared to the single-digit nanomolar and even low picomolar affinity of other clinically applied integrin-targeted radioligands, the affinities of the investigated α6-tracers were, however, fairly low (0.2–1.5 µM). Furthermore, no data on α6β4 vs. α6β1 selectivity were reported. It thus remains unclear to what extent the observed tumor uptake is causally related to expression of α6-integrins at all and, if so, to which of the two possible dimers. Improved versions of the same peptide were developed as well [128], but their affinity for α6 still remained in the micromolar range (1354 nM) and in-human data has, to the best of our knowledge, not been reported yet.

A similar situation is encountered for radiotracers targeting α5β1-integrin. The bispecific cyclic peptide c(phg-*iso*D-G-R-k) [129], which shows a high affinity for both of the functionally different integrins α5β1 [7] and αvβ6 [59], was recently labeled with ^99m^Tc via the HYNIC approach and tested for SPECT imaging of pancreatic carcinoma in humans [130]. Further studies are required to firmly establish its clinical value for cancer imaging—but the intrinsic problems of a weak biochemical rationale, i.e., the inability of the tracer to distinguish between both addressed integrins and, thus, between fundamentally different tumorigenic pathways, will remain. Interestingly, it was reported already several years ago that *N*-methylation of this peptide at the D-Lys position eliminated its αvβ6-integrin affinity, resulting in the α5β1-integrin specific peptide c(phg-*iso*D-G-R-(*N*Me)k) [131]. A respective ^68^Ga-labeled conjugate showed promise for α5β1-integrin targeted PET imaging in rodents [131], but similar to other highly specific α5β1-integrin radiotracers such as ^68^Ga-Aquibeprin [132,133], no first-in-human data were reported yet.

## 6. Conclusions

A thorough look at the latest translational research in the area of integrin-targeting radiopharmaceuticals reveals that we are currently witnessing a paradigm change. During the last two decades, the pertinent studies were strongly focused on the subtype αvβ3 and its most popular biochemical characteristics—its presence on endothelial cells and overexpression in the course of angiogenesis. It appears that the focus of interest is now moving away from αvβ3-integrin and angiogenesis towards other integrin subtypes which are involved in a large variety of tumorigenic pathways. Peptidic and non-peptidic radioligands for the integrins α5β1, αvβ6, αvβ8, α6β1, α6β4, α3β1, α4β1, and αMβ2 were synthesized and characterized in rodent models. Integrin tracers targeting the subtypes αvβ6, αvβ8, and α6β1/β4 were tested in humans. The largest number of recent first-in-human applications has been reported for radiolabeled compounds targeting αvβ6-integrin, owing to its well-established role as a biomarker for malignancy and invasiveness of carcinomas as well as its known overexpression by the most deadly cancers. Of all integrins, the subtype αvβ6 is arguably the most promising target structure for radiotheranostics, because its clinical scope is not restricted to oncological applications but also encompasses fibrotic diseases such as idiopathic pulmonary fibrosis (IPF), and probably even COVID-19 related syndromes.
